# Remission of autoimmune diabetes by anti-TCR combination therapies with anti-IL-17A or/and anti-IL-6 in the IDDM rat model of type 1 diabetes

**DOI:** 10.1186/s12916-020-1503-6

**Published:** 2020-02-28

**Authors:** Anne Jörns, Daichi Ishikawa, Hiroki Teraoku, Toshiaki Yoshimoto, Dirk Wedekind, Sigurd Lenzen

**Affiliations:** 1 Institute of Clinical Biochemistry, Hannover Medical School, Hannover, Germany; 2 Institute of Experimental Diabetes Research, Hannover Medical School, 30623 Hannover, Germany; 3 Institute for Laboratory Animal Science, Hannover Medical School, Hannover, Germany

**Keywords:** LEW.1AR1-*iddm* rat, Type 1 diabetes, Reversal of hyperglycaemia, IL-17, IL-6, Antibody combination therapy, Pancreatic beta cells

## Abstract

**Background:**

The cytokine IL-17 is a key player in autoimmune processes, while the cytokine IL-6 is responsible for the chronification of inflammation. However, their roles in type 1 diabetes development are still unknown.

**Methods:**

Therefore, therapies for 5 days with anti-IL-17A or anti-IL-6 in combination with a T cell-specific antibody, anti-TCR, or in a triple combination were initiated immediately after disease manifestation to reverse the diabetic metabolic state in the LEW.1AR1-*iddm* (IDDM) rat, a model of human type 1 diabetes.

**Results:**

Monotherapies with anti-IL-6 or anti-IL-17 showed no sustained anti-diabetic effects. Only the combination therapy of anti-TCR with anti-IL-6 or anti-IL-17 at starting blood glucose concentrations up to 12 mmol/l restored normoglycaemia. The triple antibody combination therapy was effective even up to very high initial blood glucose concentrations (17 mmol/l). The β cell mass was raised to values of around 6 mg corresponding to those of normoglycaemic controls. In parallel, the apoptosis rate of β cells was reduced and the proliferation rate increased as well as the islet immune cell infiltrate was strongly reduced in double and abolished in triple combination therapies.

**Conclusions:**

The anti-TCR combination therapy with anti-IL-17 preferentially raised the β cell mass as a result of β cell proliferation while anti-IL-6 strongly reduced β cell apoptosis and the islet immune cell infiltrate with a modest increase of the β cell mass only. The triple combination therapy achieved both goals in a complimentary anti-autoimmune and anti-inflammatory action resulting in sustained normoglycaemia with normalized serum C-peptide concentrations.

**Electronic supplementary material:**

The online version of this article (10.1186/s12916-020-1503-6) contains supplementary material, which is available to authorized users.

## Background

Type 1 diabetes (T1D) is a T cell-mediated autoimmune disease with expression and release of pro-inflammatory cytokines from pancreatic islet infiltrating immune cells, especially tumour necrosis factor alpha (TNF-α) and interleukin 1 beta (IL-1β), and other mediators, which cause selective apoptotic β cell death [1–3].

Two other pro-inflammatory cytokines, IL-17A and IL-6, have raised special interest recently. IL-17A is a pro-inflammatory cytokine produced by γ,δ T cells and CD4 T-helper cells that initiates and modulates autoimmune processes in different organs [4–9]. Specific immunomodulatory therapies by antibodies in humans to neutralize the IL-17A activating cytokine IL-23 or the effector cytokine IL-17A itself have been successful in the treatment of psoriasis, psoriatic arthritis, rheumatoid arthritis and ankylosing spondylitis [8, 10–15]. Another important cytokine produced in the infiltrating immune cells maintaining the inflammatory process is IL-6, which has been shown to induce changes in CD4 T cells in a number of autoimmune diseases [16–18]. Both IL-6 and IL-17 have also been found in the islet immune cell infiltrate of different rodent models of T1D as well as of patients with T1D [2, 19]. During disease development, IL-17A has been postulated to initiate pro-inflammatory cytokine and chemokine expression in the infiltrating immune cells [19–21], while IL-6 leads to a chronification of the inflammation in the specific organ [16–18].

Little is known about the preventive potential of a blockade of IL-17A or IL-6 by specific antibodies in T1D, both in animal models and in patients. Therefore, we analysed in the present study in the LEW.1AR1-*iddm* rat (short name: IDDM rat), a model of human T1D, after disease manifestation, the preventive potential of a rat-specific antibody against IL-17A, anti-IL-17A, which was successfully used in the therapy of experimental rheumatoid arthritis [22] and a monoclonal rat-specific antibody against IL-6, anti-IL-6, which was successfully administered in a rat hypertension model with kidney inflammation [23].

The LEW.1AR1-*iddm* rat is an animal model of human type 1 diabetes [24–26]. It is one of three established rat models with a MHC class II *u* haplotype with an average disease manifestation around 60 days of life and an incidence of around 50% with disease characteristics closely resembling those of human T1D [27]. A point mutation responsible for diabetes manifestation resides in the *Dock8* gene [28].

In this rat model of human T1D [24, 26], both cytokine antibodies against IL-17A and IL-6 were administered alone as done also with an antibody against the rat pro-inflammatory cytokine TNF-α [25]. Furthermore, we combined an antibody against TCR, which alone also provided no sustained therapy success [25, 29] with anti-IL-17A or with anti-IL-6. In addition, we also analysed here for the first time a triple combination of anti-TCR with anti-IL-17A plus anti-IL-6 to overcome limitations in therapeutic effectiveness of each of these antibodies alone.

The results with this triple combination convincingly document the pronounced β cell restorative potential of this new antibody therapy for reversal of the diabetic metabolic state and thus confirm the need for combination therapies to successfully target pro-inflammatory mediators produced in the islet infiltrating immune cells and to restore normoglycaemia [2, 30–34].

## Methods

### Animals

Congenic IDDM rats (for details see http://www.mh-hannover.de/34926.html) were bred through mating of diabetic female and male rats under specific pathogen-free (SPF) conditions and thereafter housed under standard conditions in the Central Animal Facility of Hannover Medical School as described [24, 25, 29]. They were serologically negative for specific viruses and regularly submitted to genetic monitoring. Two pancreas tissue biopsies (removal of 30 mg pancreas each from the pancreas tail) were performed as described in detail before at the day of diabetes manifestation before start of therapy and after the end of the 5-day therapy [25, 29]. Experimental procedures were approved by the District Government of Hannover (LAVES, No 33-42502-05/958 & 509.6-42502-03/684 and 33.9-42502-04/16/2115) in accordance with the guide for the care and use of laboratory animals.

### Experimental groups

Nine experimental groups of IDDM rats of both sexes were studied comprising a total of 55 animals. Group 1 (*n* = 6) comprised healthy, normoglycaemic IDDM rats which received 0.9% NaCl solution only, group 2 (*n* = 6) comprised acutely diabetic IDDM rats which received IgG (I5381; Merck, Darmstadt, Germany; diluted with 0.9% NaCl solution) and groups 3, 4 and 5 (*n* = 4 each) comprised acutely diabetic IDDM rats treated for 5 consecutive days in a monotherapy either with a rat-specific anti-TCR antibody (Clone: R73; Bio-Rad, Munich, Germany) (0.5 mg/kg b.wt. i.v.), rat-specific anti-IL-6 (0.01 mg/kg b.wt. i.v.) (Clone: MAB 561**,** R&D Systems, Wiesbaden-Nordenstadt, Germany) or with a murine specific anti-IL-17A (0.1 mg/kg b.wt. i.v**.**) (Clone: 17F3, eBioscience, Frankfurt, Germany**)**. Group 6 comprised a combination of anti-IL-6 plus anti-IL-17A (*n* = 4). Only antibodies against IL-17A, not anti-IL-17F, are effective [35, 36]. The other treatments for 5 consecutive days were combination therapies of anti-TCR with anti-IL-6 in group 7 (*n* = 7) or with anti-IL-17A in group 8 (*n* = 10) in the same doses as mentioned above. In addition, a triple combination of anti-TCR with anti-IL-6 plus anti-IL-17A was analysed in group 9 (*n* = 10). The different therapies were identical in all rats in each group and were started within 1 day after diabetes onset at blood glucose concentrations > 7.5 mmol/l.

### Tissue processing

Tissue specimens were fixed for microscopic analyses [29]. Blood glucose concentrations were determined daily (Glucometer Elite®, Bayer, Leverkusen, Germany). Serum C-peptide was analysed with a rat-specific ELISA (Mercodia, Uppsala, Sweden), and serum cytokine protein concentrations with a multiplex immunoassay kit (Bio-Rad) [25, 29].

### Morphological analyses

Serial sections stained either with the avidin-biotin-complex method or the double immunofluorescence technique were performed with primary antibodies for β cells and immune cells provided in Additional file 1: Table S1 [2, 25] and the secondary antibodies in Additional file 1: Table S2. The antibodies against IL-6 (polyclonal goat; #500-P73G, PeproTech, Hamburg, Germany), IL-17A (polyclonal rabbit, sc-7927, Santa Cruz Biotechnology, Heidelberg, Germany) and α,β CD3 T cells (1F4, Bio-Rad) recognized epitopes other than those targeted by the treatment antibodies. In addition, a specific staining for γ,δ T cells was performed (V65, MCA1146, Bio-Rad). The immunohistochemical stainings were checked by replacement of the primary or secondary antibodies by buffer showing no specific staining. Β cell proliferation or apoptosis with a minimum of 1000 β cells, β cell mass and the insulitis score as well as cytokine and cell cycle marker gene expression were quantified as described using the Olympus microscopes BX61 and for scanning BX61VS (Olympus, Hamburg, Germany) [2, 25, 29].

### In situ reverse transcriptase-polymerase chain reaction (in situ RT-PCR)

In situ RT-PCR analyses were performed on pancreatic sections placed on three-chamber slides from all experimental groups. The procedure was performed on a special thermal cycler (Bio-Rad) as described [25, 29]. The used primer sequences with the accession numbers are provided in Additional file 1: Table S3. Control experiments for the PCR reaction performed without primers showed no specific staining in the respective cell types.

### Statistical analyses

Results are presented as mean values ± SEM. Numbers of rats and numbers of experiments are identical during the observation period before, after and 60 days after the end of therapy. Comparisons between the different therapy groups and the normoglycaemic or diabetic controls were analysed with ANOVA followed by Dunnett’s test or Bonferroni’s test for multiple comparisons with the Prism 5 programme (GraphPad Inc., San Diego, CA). Significance was accepted at *p* < 0.05.

## Results

### Metabolic effects of therapy with anti-IL-6 or anti-IL-17A alone and in combination with anti-TCR in a double or triple fashion

**Fig. 1:**
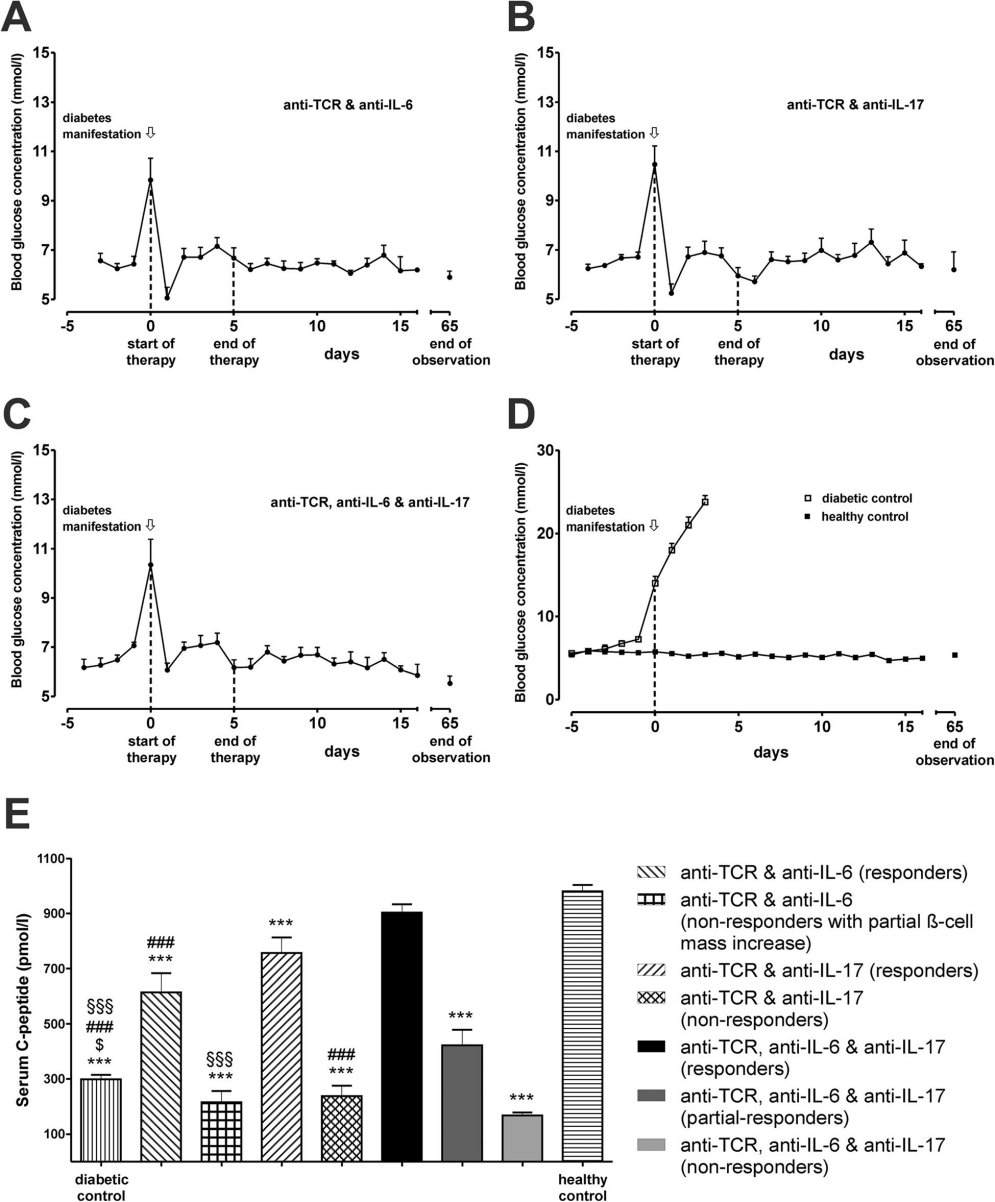
Effects of anti-TCR combination therapies on the metabolic profile of IDDM rats after diabetes manifestation**. a–d** Blood glucose concentration (mmol/l) changes are shown for the responding rats in response to the different anti-TCR combination therapies **a** with anti-IL-6 (5/7), **b** anti-IL-17A (7/10) or **c** in the triple combination (6/10) compared to **d** the normoglycaemic healthy (*n* = 6) and to the acutely diabetic untreated IDDM rats (*n* = 6). The first dashed line at day 0 indicates the start of therapy (first biopsy) and the second dashed line at day 5 indicates the end of therapy (second biopsy). **e** Serum C-peptide concentration changes (pmol/l) are shown for rats responding and non-responding to the different combination therapies of anti-TCR with anti-IL-6 or anti-IL-17A alone or in combination. Data are mean values ± SEM. Comparison of the different experimental groups by one-way ANOVA followed by Bonferroni test ***p < 0.001 to the healthy control, ^$^*p* < 0.05 to anti-TCR combination with anti-IL-6, ^§§§^*p* < 0.001 to anti-TCR combination with anti-IL-17A, and ^###^*p* < 0.001 to triple combination for each observation time point

Anti-IL-6 (0.01 mg/kg b.wt., for 5 days) or anti-IL-17A (0.1 mg/kg b.wt., for 5 days) were administered to IDDM rats within 1 day after diabetes manifestation (blood glucose > 7.5 mmol/l), either alone or in combination with anti-TCR (0.5 mg/kg b.wt., for 5 days) in a double or triple fashion. Anti-TCR therapy in combination with anti-IL-6 (Fig. 1a) or anti-IL-17A (Fig. 1b) resulted after both therapies in a return to normoglycaemia (defined as blood glucose < 7.0 mmol/l in comparison to the diabetic and the normoglycaemic healthy control animals) (Fig. 1d) after a 60-day therapy-free interval following the end of the 5-day antibody therapy course of the rats for anti-IL-6 (5 out of 7 rats) (Fig. 1a) and for anti-IL-17A (7 out of 10 rats) (Fig. 1b). The improvement was particularly pronounced after the triple combination treatment (6 out of 10 rats for anti-IL-6 and anti-IL-17A) reaching a mean blood glucose value of 5.8 mmol/l at day 65 (Fig. 1c) being not significantly different from the mean blood glucose value of 5.4 mmol/l at the same time point in the healthy control rats (Fig. 1d). In contrast, diabetic rats without therapy remained severely hyperglycaemic (Fig. 1d). No adverse events were observed during and after the treatment.

In the successfully treated animals with the different anti-TCR combination therapies with anti-IL-6 or anti-IL-17A alone or in a triple fashion serum C-peptide concentrations increased (Fig. 1e). Sixty days after the end of therapy, C-peptide values were more than doubled (*p* < 0.01) compared to the values of the diabetic animals (ranging between 200 and 300 pmol/l) before the start of therapy.

Improvement of C-peptide values was better in the combination with anti-IL-17A (757 ± 57 pmol/l) than with anti-IL-6 (615 ± 68 pmol/l). The greatest improvement of C-peptide values was achieved with the triple combination reaching values (903 ± 30 pmol/l) that were close to the C-peptide concentrations in the healthy control rats (981 ± 22 pmol/l) (Fig. 1e).

**Fig. 2:**
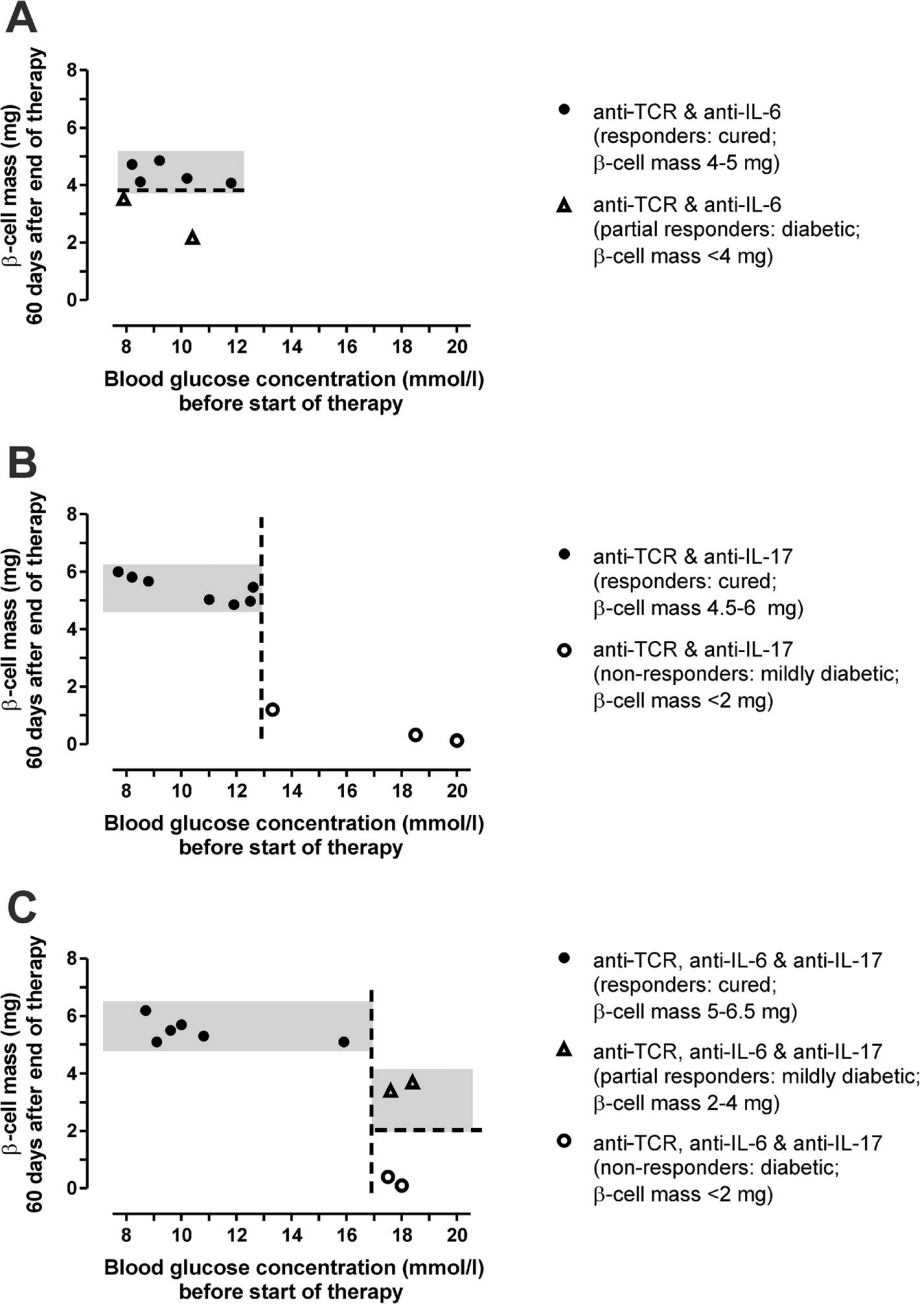
Relation between initial blood glucose concentration and β cell mass after end of therapy. **a** After anti-TCR combination therapy with anti-IL-6 or **b** with anti-IL-17A or **c** with both cytokine antibodies together. The β cell mass showed the highest values after triple combination, followed by the double combination with anti-IL-17A and the lowest values after double combination with anti-IL-6. Remarkably, the starting blood glucose concentrations granting therapy success differed between the three analysed groups without a blood glucose concentration window for anti-TCR combination with anti-IL-6 and with starting blood glucose concentrations below 13 mmol/l for the anti-TCR combination with anti-IL-17A and 17 mmol/l for the triple combination compared to those above these glucose values

The different combination therapies were initiated within 1 day after diabetes onset at blood glucose concentrations > 7.5 mmol/l. A detailed analysis revealed interesting results regarding therapy success as documented by an increased β cell mass approaching the control values of the non-diabetic animals (around 6 mg) in relation to the initial blood glucose concentrations (Fig. 2). The exception was observed after the anti-TCR combination therapy with anti-IL-6 where maximal β cell mass values of no more than 4 mg were achieved even at moderately increased starting blood glucose concentrations in the diabetic rats and even this modest improvement was not achieved in all treated animals (Fig. 2a).

In the double combination therapy group of anti-TCR with anti-IL-17A, β cell mass values were raised without exception to values in the range between 5 and 6 mg when treatment was initiated at blood glucose values below 13 mmol/l (Fig. 2b). At higher starting blood glucose values therapy was unsuccessful (Fig. 2b).

In the triple combination therapy of anti-TCR with anti-IL-6 plus anti-IL-17A, therapy was successful at all starting blood glucose concentrations below 17 mmol/l, reaching again β cell mass values in the range between 5 and 6 mg, which were comparable to those in healthy control rats (Fig. 2c).

Animals responding with a partial increase of the beta cell mass after treatment with anti-TCR plus anti-IL-6 (Fig. 2a) were nevertheless unable to achieve sustained normoglycaemia. Though therapy was initiated at a blood glucose concentration of 9.2 ± 1.3 mmol/l (*n* = 2) and ended after 5 days of therapy at a blood glucose concentration of 8.2 ± 0.4 mmol/l (*n* = 2), the blood glucose concentration 60 days after therapy was in the hyperglycaemic range with a value of 19.6 ± 1.7 mmol/l (*n* = 2) due to an insufficient maintenance of the insulin secretory capacity of the beta cells as documented by the fact that the serum C-peptide levels did not increase above levels in diabetic animals (Fig. 1e). Thus, these animals are depicted as non-responders with respect to C-peptide levels (Fig. 1e) and depicted as partial responders with respect to a limited beta cell mass increase (Fig. 2a).

This is in contrast to the partial responders in the triple combination treatment (anti-TCR plus anti-IL-6 and anti-IL-17), where the glucose concentrations showed a partial reduction of hyperglycaemia from an initial high blood glucose concentration of 18.1 ± 0.3 mmol/l (*n* = 2) to a value to 9.1 ± 0.2 mmol/l (*n* = 2) and a value of 12.3 ± 3.3 mmol/l (*n* = 2) 60 days after therapy along with a partial increase of the beta cell mass (Fig. 2c) and a partial increase of the C-peptide levels (Fig. 1e).

In rats (*n* = 4 in each group) with monotherapies with anti-TCR, anti-IL-6, or anti-IL-17 as well as the combination of anti-IL-6 plus anti-IL-17 (Additional file 2: Figures S1 and Additional file 3: Figure S2) in a fashion analogous to the double and triple combinations with anti-TCR (Figs. 1 and 2), no therapy success could be achieved after diabetes manifestation with blood glucose concentrations remaining in a permanent hyperglycaemic state (> 15.0 mmol/l) during and up to the end of the observation period of 10 days after start of the treatment (Additional file 2: Figure S1a-d). All treatments were also completely ineffective in raising C-peptide concentrations above levels of diabetic animals (Additional file 2: Figure S1e). The beta cell mass of all animals in these groups did not respond to treatment remaining in a very low range in all groups (< 0.5 mg) typical for diabetic animals (Additional file 3: Figure S2a-d).

### Morphometric quantification of therapeutic effects on β cells and pancreatic islet infiltration after combination therapies

#### Changes of proliferation and apoptosis rates in β cells

**Fig. 3:**
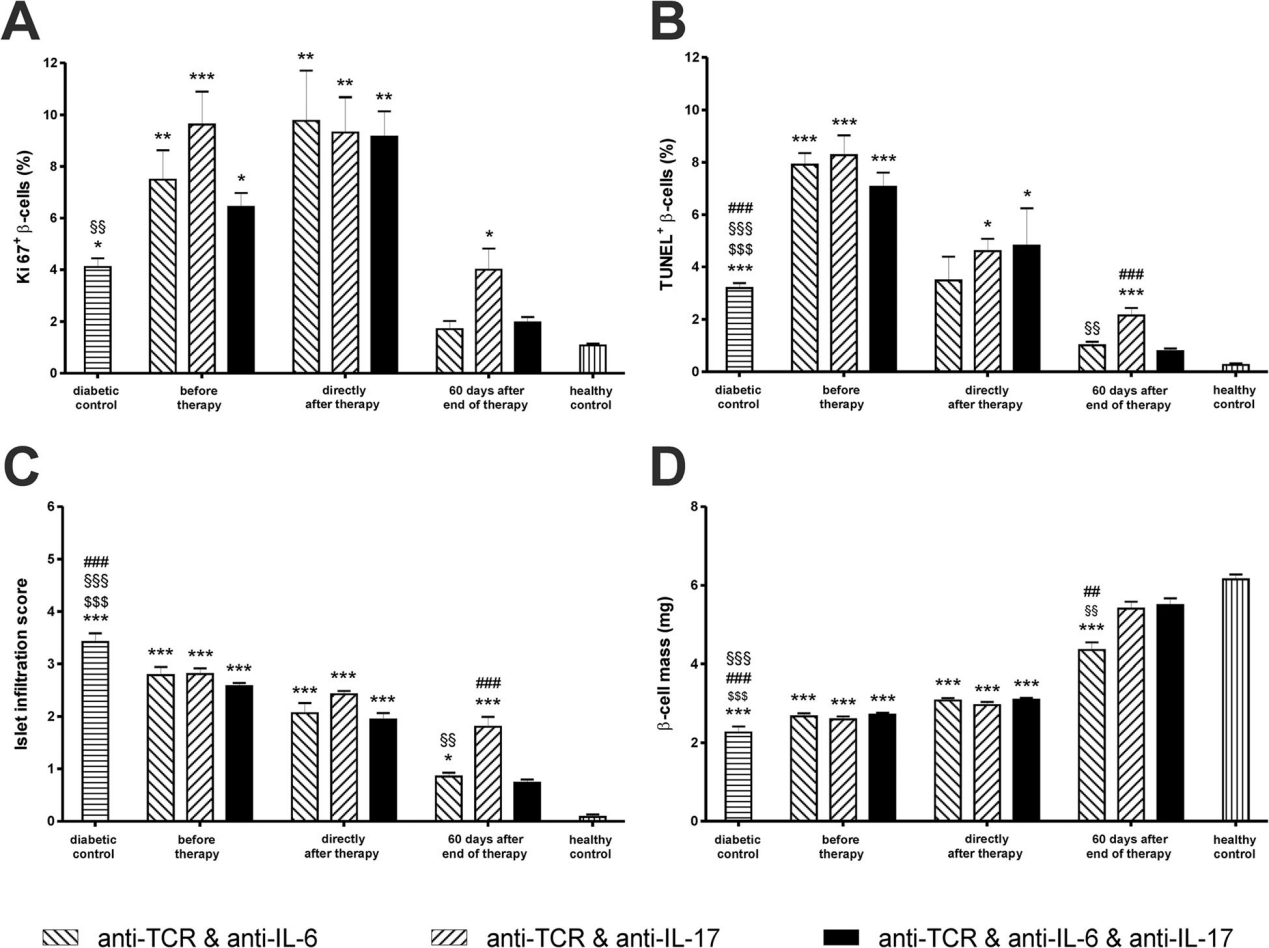
Morphometric analyses of β cells and immune cells in IDDM rats after anti-TCR combination therapies. Changes in the rate of **a** proliferation, **b** apoptosis, **c** islet infiltration score and **d** pancreatic β cell mass after successful anti-TCR combination therapy with anti-IL-6 or anti-IL-17A alone or combined together after diabetes manifestation. Measurements were performed immediately before therapy (first biopsy), at the end of therapy (second biopsy) and 60 days after the end of therapy Data are mean values ± SEM. Comparison of the different experimental groups by one-way ANOVA followed by Bonferroni test ***p < 0.001, **p < 0.01 and *p < 0.05 to the healthy control, ^$$$^*p* < 0.001 to anti-TCR combination with anti-IL-6, ^§§§^*p* < 0.001 and ^§§^*p* < 0.01 to anti-TCR combination with anti-IL-17A, ^###^*p* < 0.001 and ^##^*p* < 0.01 to triple combination for each observation time point. Numbers of pancreases analysed as given in Fig. 1

At the day of diabetes manifestation, immediately before the start of therapy at the time point of the first biopsy, the rats in the therapy groups with anti-TCR in combination with anti-IL-6 and with anti-IL-17 as well as with both anti-cytokine antibodies showed significant 6–9-fold increases of the proliferation rates analysed by Ki67 staining compared to normoglycaemic controls (Fig. 3a). The apoptosis rates analysed by TUNEL increased 24–28-fold compared to normoglycaemic controls (Fig. 3b).

Immediately after the end of the different combination therapies at the time point of the second biopsy, only the anti-TCR combination therapy together with anti-IL-6 or with anti-IL-6 plus anti-IL-17A showed a further increase of the proliferation rate (Fig. 3a). In all anti-TCR combination therapies with anti-IL-6 or anti-IL-17A alone or with both antibodies the apoptosis rate was reduced by around 50% already at the end of the 5-day therapy (Fig. 3b).

At 60 days after the end of therapy, rats successfully treated with the combination of anti-TCR with anti-IL-6 alone or in the triple therapy showed still a slight doubled proliferation rate whereas in the anti-TCR combination with anti-IL-17A the proliferation rate remained significantly increased by a factor of 4 as compared to healthy controls (Fig. 3a). In the triple combination therapy or in the anti-TCR combination with anti-IL-6 the apoptosis rate was no more significantly increased when compared to healthy controls whereas the anti-TCR combination with anti-IL-17A showed still a significantly increased apoptosis rate compared also to the triple combination (Fig. 3b).

#### Changes in proliferation/apoptosis ratios in β cells

Calculation of the proliferation/apoptosis ratios revealed a decrease to half of the values of the healthy controls in both double combination therapies; only in rats responding to the triple combination therapy the ratio was identical to that in the healthy controls (Additional file 1: Table S4).

#### Infiltration score

On the day of diabetes manifestation, before the start of therapy, the infiltration score of the islets was high with values above 2.5 for all combination therapies (Fig. 3c). The insulitis score was not significantly reduced after the end of all combination therapies (Fig. 3c). At 60 days after the end of therapy, the infiltration score in the regenerated endocrine pancreases was reduced to values < 1.0 for the anti-TCR combination with anti-IL-6 as well as the triple combination and < 2.0 for the anti-TCR combination with anti-IL-17A (Fig. 3c).

#### β cell mass

On the day of diabetes manifestation, before the start of therapy, the β cell mass of the pancreases was reduced in all diabetic rats to around 1/3 of the value in the controls (Fig. 3d). Immediately after the end of the anti-TCR combination therapies with anti-IL-6 and anti-IL-17A alone or in the triple fashion, the pancreatic β cell mass was moderately increased to half of the values in healthy control rats (Fig. 3d). Sixty days after the end of the different combination therapies with anti-IL-17A, the β cell mass had attained values in the normal range (Fig. 3d) whereas the β cell mass in the anti-TCR combination with anti-IL-6 reached only around 2/3 of the control values (Fig. 3d).

In summary, thus when compared to the pre-treatment situation, the β cell mass of the pancreases (Fig. 3d) was increased and the rates of proliferation, apoptosis and islet infiltration were reduced (Fig. 3a–c) 60 days after the end of therapy to levels comparable to the healthy control situation. On the other hand, in the monotherapy treatment groups, positive effects were observed at the end of the observation period neither on the mass of the beta cells nor on proliferation, apoptosis and insulitis score (Additional file 4: Figure S3a-d). This is in clear contrast to the positive effects of the combination therapies presented in Fig. 3.

### Effects of therapies on the immune cell infiltration pattern in pancreatic islets

**Fig. 4:**
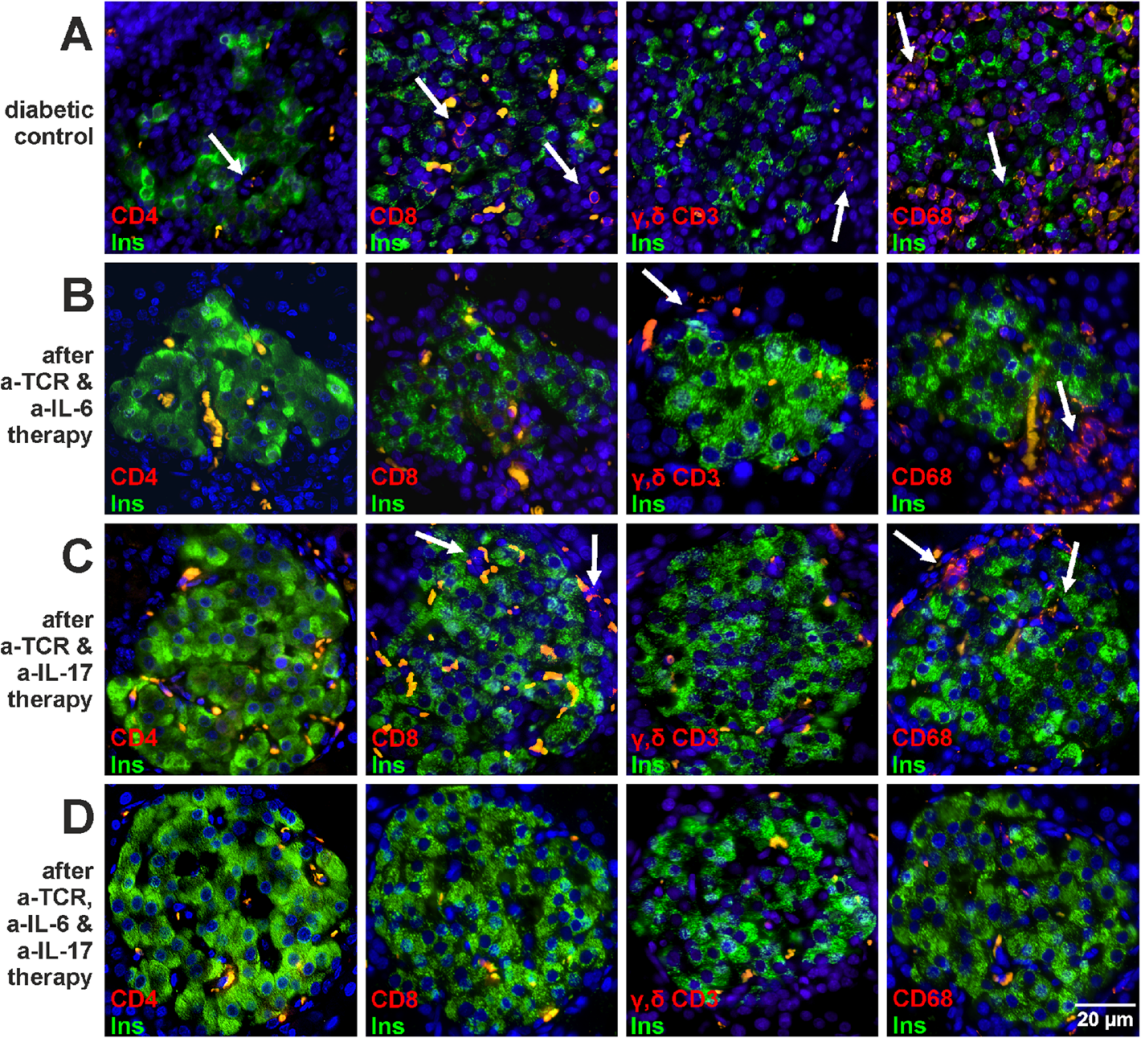
Immune cell infiltration in pancreatic islets of IDDM rats after successful anti-TCR combination therapies. **a–d** β Cells (*green*) and immune cells (*red*) were examined in islets from animals successfully treated with anti-TCR and anti-IL-6 (**b**) or with anti-TCR and anti-IL-17A (**c**) or with anti-TCR and both cytokine antibodies (**d**) after diabetes manifestation and compared to the untreated diabetic situation (**a**). Islets were immunostained for insulin (*green*) and CD68 macrophages (*red*), CD8 T cells (*red*), or γ,δ T cells (red) and counterstained with DAPI (*blue*). Erythrocytes were identified by *yellow* to *orange* colour through auto-fluorescence in the red and green channel. In each group, 40 to 80 islets in the pancreases were analysed

In the diabetic control rats, the islet infiltrate (Fig. 4a) was composed of around 37% CD8 T cells and 45% CD68 macrophages, with a smaller amount of 4% CD4 and 4% of γ,δ T cells each as well as 10% of other immune cell types in the acutely diabetic rats (Table 1).

**Table 1:** Presence of different immune cell types in the pancreatic islets

Cell types	Healthy control (n/mm^2^)	Anti-TCR and anti-IL-6 (n/mm^2^)	Anti-TCR and anti-IL-17A (n/mm^2^)	Anti-TCR, anti-IL-6 and anti-IL-17A (n/mm^2^)	Diabetic control (n/mm^2^)
CD4 T cells	0 ± 0	0 ± 0	1 ± 0*	0 ± 0**	4 ± 0
CD8 T cells	0 ± 0	0 ± 0**	5 ± 1**	0 ± 0**	26 ± 3
γ,δ T cells	0 ± 0	2 ± 1	0 ± 0**	0 ± 0**	3 ± 1
CD68 MØ	3 ± 1	7 ± 1**	8 ± 0**	3 ± 1**	34 ± 4
Others	0 ± 0	0 ± 0	0 ± 0	0 ± 0	8 ± 2

Quantification of the different immune cell types in the pancreatic islet after immunostaining for CD4, CD8, γ,δ T cells and CD68 macrophages 60 days after the end of different anti-TCR combination therapies with anti-IL-6 or anti-IL-17A alone or in triple fashion in the LEW.1AR1*-iddm* rats. The quantitative analysis of the frequency in the immune cells was performed in the pancreases of the successfully treated rats in all groups (a total of 50 to 80 islets in each group. *p < 0.05, **p < 0.01 by ANOVA followed by Dunnett’s test for all successful therapies against the diabetic controls. Numbers of pancreases analysed as given in Fig. 1

Sixty days after the end of the different combination therapies, islet immune cell infiltration in the responding rats was very markedly reduced (Fig. 4b–d and Table 1). After anti-TCR combination therapy with anti-IL-6, only a very few γ,δ T cells and CD68 macrophages were still residing in the periphery of the islets (Fig. 4b and Table 1). After anti-TCR combination with anti-IL-17A, very few CD4 T cells and some CD8 T cells and CD68 macrophages were still visible inside and around the islets along with a complete absence of γ,δ T cells (Fig. 4c and Table 1). In view of this islet immune cell infiltration, the insulitis score (Fig. 3c) remained distinctly higher in the combination with anti-IL-17A than with anti-IL-6. The triple combination resulted in contrast to the double combination therapies in a complete disappearance of all T cell subtypes in islets (Fig. 4d and Table 1). Only very occasionally single infiltrating CD68 macrophages were still detectable in the islets after triple combination therapy in a quantity identical to the small number of in the healthy control pancreas (Table 1). These CD68 macrophages are not activated (i.e. not a site of pro-inflammatory cytokine production) and act as scavengers for cell debris.

### Changes in the gene expression pattern of cytokines in pancreatic islets after combination therapies

Immune cells infiltrating the islets were activated in diabetic rats with blood glucose concentrations > 15 mmol/l without antibody therapies. This was documented by high gene expression levels of the pro-inflammatory cytokines, *Tnf*, *Il1b*, *Ifng*, *Il2*, *Il6* and *Il17* as well as the anti-inflammatory cytokines *Il4* and *Il10* in the infiltrating immune cells (Table 2). Sixty days after the end of anti-TCR combination therapy with anti-IL-6 or anti-IL-17A or in the triple fashion with both antibodies, the pro-inflammatory cytokine gene expression was strongly reduced. The combination of anti-TCR with anti-IL-6 was clearly more effective than the combination with anti-IL-17A. However, only the triple combination abolished the diabetes induced pro-inflammatory cytokine increases completely, identical with the situation in islets from healthy control animals (Table 2). Interestingly, expression of the anti-inflammatory cytokines *Il4* and *Il10* was present still at a low level after combination of anti-TCR with anti-IL-17A (Table 2). The same was true for a significant expression of the anti-inflammatory cytokine *Il10* after combination of anti-TCR with anti-IL-6 and anti-IL-17A (Table 2).

**Table 2:** Pro-and anti-inflammatory cytokine gene expression by in situ RT-PCR in the islet immune cell infiltrate

Gene	Healthy control	Anti-TCR and anti-IL-6	Anti-TCR and anti-IL-17A	Anti-TCR, anti-IL-6 and anti-IL-17A	Diabetic control
TNF	0.0 ± 0.0	1.1 ± 0.1**	5.3 ± 0.3**	0.0 ± 0.0**	19.4 ± 0.6
IL1B	0.1 ± 0.0	0.8 ± 0.1**	6.8 ± 0.4**	0.0 ± 0.0**	23.7 ± 1.0
IFNG	0.0 ± 0.0	0.2 ± 0.1**	0.4 ± 0.1**	0.0 ± 0.0**	1.7 ± 0.2
IL2	0.0 ± 0.0	0.4 ± 0.1	0.8 ± 0.1	0.0 ± 0.0	2.8 ± 0.2
IL4	0.0 ± 0.0	0.1 ± 0.1	1.1 ± 0.1	0.0 ± 0.0	5.9 ± 0.4
IL10	0.0 ± 0.0	0.4 ± 0.1	0.9 ± 0.1	1.5 ± 0.3	3.1 ± 0.3
IL6	0.0 ± 0.0	0.0 ± 0.0**	2.0 ± 0.2*	0.0 ± 0.0**	5.6 ± 0.2
IL17A	0.0 ± 0.0	1.4 ± 0.2**	0.0 ± 0.0**	0.0 ± 0.0**	4.0 ± 0.2

Gene expression changes for the pro- and anti-inflammatory cytokines TNF (*Tnf)*, IL1B (*Il1b)*, IFNG (*Ifng*), IL2 (*Il2*), IL6 *(Il6)*, IL17A *(Il17a)*, IL4 (*Il4*) and IL10 (*Il10*) in the immune cells infiltrate of pancreatic islet 60 days after the end of different anti-TCR combination therapies with anti-IL-6 or anti-IL-17A alone or in triple fashion in the LEW.1AR1*-iddm* rats. Quantitatively, a percentage of the immune cell infiltrate area showing a positive mRNA transcript for the different cytokines was calculated in the pancreases of the successfully treated rats in all groups (a total of 50 to 80 islets in each group). *p < 0.05, **p < 0.01 by ANOVA followed by Dunnett’s test for all successful therapies against the diabetic controls. Numbers of pancreases analysed as given in Fig. 1

### Changes of pro- and anti-inflammatory cytokines in the circulation after combination therapies

**Fig. 5:**
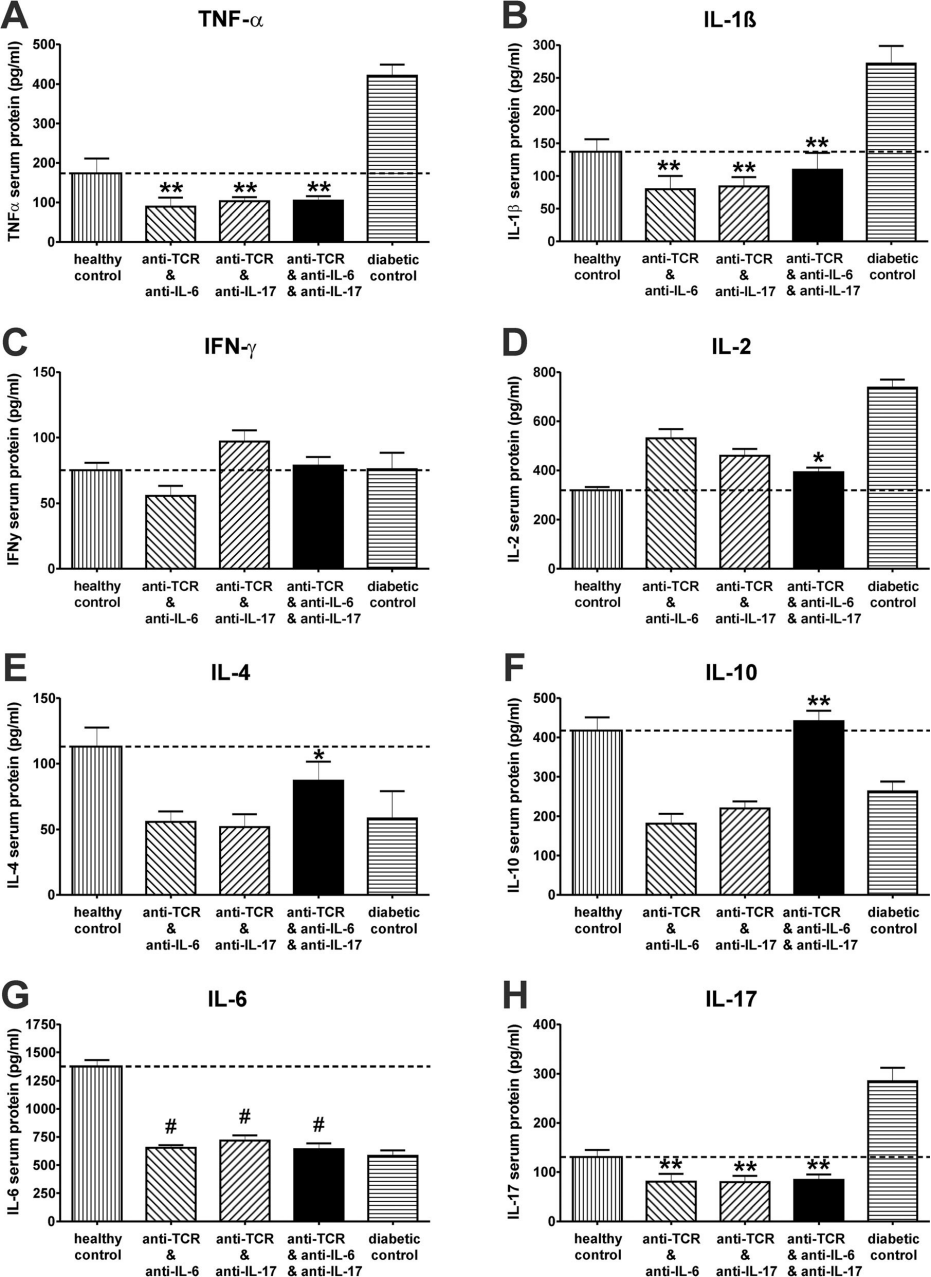
Cytokine pattern in serum of IDDM rats after successful anti-TCR combination therapies. Changes in protein concentrations of cytokines measured by multiplex analysis were examined after successful anti-TCR combination therapy with anti-IL-6 or anti-IL-17A alone or in triple combination after diabetes manifestation **a** TNF-α, **b** IL-1β, **c** IFN-γ, **d** IL-2, **e** IL-4, **f** IL-10, **g** IL-6 and **h** IL-17A. Results after anti-TCR combination therapy with anti-IL-6, with anti-IL-17A and in the triple fashion were compared to those from healthy controls and untreated, acutely diabetic rats. Cytokine protein concentrations (pg/ml) are expressed as mean values ± SEM; **a–h** the dotted lines show changes in the pro-inflammatory and anti-inflammatory cytokines compared to the normoglycaemic situation in healthy control rats. *p < 0.05 by ANOVA followed by Dunnett’s test versus untreated, diabetic controls **p < 0.01 versus untreated, diabetic controls. Number of animals as given in Fig. 1

In the animals responding to double and triple combination therapies, the serum protein concentrations of the pro-inflammatory cytokines, TNF-α and IL-1β, were 60 days after the end of the therapies as low as in healthy control animals with a significant decrease compared to the diabetic controls (Fig. 5a, b). The pro-inflammatory cytokine interferon gamma (IFN-γ) showed the same values in all groups including the control groups (Fig. 5c). The increased levels of the immune cell activating cytokine IL-2 in diabetic control rats decreased in all animals after the different combination therapies but with a significant reduction only in the triple combination compared to the diabetic controls (Fig. 5d). Only after triple combination therapy of anti-TCR with anti-IL-6 and anti-IL-17A the protein concentration of the anti-inflammatory cytokine IL-4 was somewhat increased (Fig. 5e). The anti-inflammatory cytokine IL-10 increased even to values comparable to those in healthy controls after triple combination therapy (Fig. 5f). The serum concentrations of IL-6 and IL-17A decreased after double and triple combination therapies to a range not higher than in healthy controls (Fig. 5g, h).

## Discussion

We initially compared the effectiveness of a combination of anti-TCR (analogous to anti-CD3 in humans and mice) and anti-IL-6 as well as a combination of anti-TCR and anti-IL-17A in diabetic rats. The prominent curative element of the combination of anti-TCR and anti-IL-17A was the strong β cell regenerative potential of this therapy, while the prominent curative element of the combination of anti-TCR and anti-IL-6 was the virtually complete abolishment of the autoimmune-mediated inflammatory component in the islet infiltrate. However, only the triple combination therapy of anti-TCR and anti-IL-17A plus anti-IL-6 connected both curative elements in a perfect complimentary manner. In contrast, monotherapies with anti-IL-6 or anti-IL-17A alone as well as a combination of both cytokine antibodies showed no significant potential for a reversal of the diabetic state, even when blood glucose concentrations were only minimally increased at the start of therapy. So the present studies provide additional proof for the ineffectiveness of antibody monotherapies in the T1D setting [25, 29, 33, 34, 37–39]. This is not surprising because the therapy goal in T1D is a complete halt of the autoimmune and inflammatory process in the islets, since it is the only way to allow effective β cell regeneration and a regain of normal blood glucose concentrations. The underlying mechanism of immune cell or cytokine antibody therapy is the blockade of either the target on the specific cell type surface or the specific cytokine. This T1D therapy scenario is at variance from other autoimmune diseases such as rheumatoid arthritis, intestinal bowel diseases, psoriasis and atopic dermatitis, in which the more modest therapy goal, namely a symptom free remission, can be achieved in many cases by a monotherapy, for example with anti-TNF or anti-IL-17A [12, 40–42]. This, however, is not a feasible therapy approach in T1D, where the only option is a complete eradication of the β cell destructive immune cell infiltration process, because only this permits the restoration of a sufficient β cell mass as a prerequisite for achieving a sustained normoglycaemia by a combination of different immunomodulatory agents, as it is consensus in the scientific community [2, 30–34]. Looking at the initial β cell mass at the start of therapy, it can be concluded that a residual β cell mass of around one third of that in the healthy control situation is what is needed for successful restoration of sustained normoglycaemia along with C-peptide and β cell mass values in the normal range [31, 34].

Immunomodulatory antibody therapy already significantly reduced the β cell apoptosis rate during the 5-day therapy interval as documented by pancreas biopsies of the rats before and immediately after the end of therapy. Only during the subsequent 60-day observation period after the end of the antibody therapy, however, the large increase of the β cell mass was achieved as a result of effective β cell proliferation along with low apoptosis rates. The β cell proliferative process was mainly mediated by the IL-17A antibody administration while the IL-6 antibody in spite of its strong anti-inflammatory effect only induced a moderate β cell mass increase. Maximal was only the effect of a triple combination therapy, restoring β cell mass with an increase to values drawing near those of the healthy animal. The positive effect on β cell regeneration using anti-IL-17A in the process of disease reversal has been unknown until now. The triple antibody combination has not yet been used in the treatment of any other autoimmune disease [34, 41, 42]. However, the mutual interaction between IL-6 and IL-17A for differentiation of specific T cells is known from other inflammatory processes [17, 43].

Confirming earlier observations in other diseases, the pro-inflammatory interleukin IL-6 was expressed in all immune cell subtypes [16–18, 44] during the inflammation process in the T1D pancreas, while IL-17A was only expressed in the subgroup of the γ,δ T cells and CD4 T helper cells [4–6, 9] comprising about 10% of the immune cells in the pancreatic islet. The specific antibody each completely suppressed the expression of the respective interleukin (IL-6 as well as IL-17A) in the islets as documented by the in situ PCR analyses in the present study. In the case of IL-17A, such a reduction has also been shown during treatment of other autoimmune diseases, such as psoriasis, rheumatoid arthritis and multiple sclerosis [45–48]. The same was true for changes in serum protein levels of IL-6 in rheumatoid arthritis [45, 49]. The gene expression of the other interleukin each was also significantly reduced. Increases of anti-inflammatory cytokines (i.e. IL-4, IL-10) were also dampened. But noticeably expression of IL-10 remained clearly present, in particular after the triple combination therapy. A complete abolishment of the expression of the pro-inflammatory cytokines (i.e. TNF-α, IL-1β) in the islet infiltrate was achieved only by the triple antibody combination. This explains why the triple combination, but not the double combinations provided the desired full therapy success. The monotherapies were ineffective in this respect. Crucial for this sustained therapy success was the total suppression of pro-inflammatory cytokine gene expression in the islets along with the total disappearance of T cell infiltration thereby preventing the β cell toxic effect of the pro-inflammatory cytokines [50, 51] responsible for β cell loss in the T1D pancreas [25].

In the serum cytokine protein profile, the two main pro-inflammatory cytokines, TNF-α and IL-1β, decreased whereas the anti-inflammatory cytokines, IL-4 and IL-10, increased to values of the normoglycaemic controls in the same way as described for the therapy success after the combination with anti-TCR and anti-TNF-α in the rat model [25] and in the human situation [52]. High levels of IL-10 are considered to be important for long-term protection and preservation of β cell function [53] and T1D prevention [54]. Though not expressed in islets of control animals, serum levels of IL-17A and in particular of IL-6 were high in healthy control animals, providing evidence for the presence of sources in the organism other than the islets [44, 55]. These serum levels decreased after administration of the respective antibodies as also observed before in the treatment of other autoimmune diseases [45–48].

It can be concluded from the present studies that a β cell mass and C-peptide restoration to an extent allowing the achievement of a sustained normoglycaemia along with an infiltration-free endocrine pancreas was best achieved with the triple antibody combination.

The results obtained with the two double antibody combination therapies showed that this full therapy success of the triple combination was the result of two different therapeutic effects, namely a strong β cell proliferative effect optimally supporting the β cell mass increase of anti-IL-17A and a strong anti-apoptotic and anti-inflammatory effect of anti-IL-6, achieved in the combination with anti-TCR (analogous to anti-CD3 in humans and mice), an antibody directed against the TCR/CD3 complex, which is a cornerstone compound in anti-T cell therapy [56]. The dissociation between these two effects is a novel observation and shows for the first time, that it is necessary for an optimal therapeutic effect to successfully target both the autoimmune and the inflammatory components in the T1D disease process. Thus, these two double combination therapies provide an interesting research tool to elucidate the roles of the differential effects required for an optimal outcome of a therapy with full curative potential.

## Conclusions

In conclusion, the triple antibody combination was the most successful combination, which we have ever characterized in the IDDM rat model of human T1D [26]. This result has even been achieved at starting blood glucose values above 10 mmol/l and up to values of 17 mmol/l, a result which we were not able to achieve with any other combination therapy (i.e. anti-TCR plus anti-TNF-α) [25]. This triple combination therapy kept the therapeutic window open for a long time after disease manifestation. Nevertheless, the optimal time point for initiation of curative therapies is always the time immediately (as soon as possible) after disease manifestation.

In summary, the results of this study document convincingly the effectiveness of curative combinatory antibody therapeutic approaches, which target pro-inflammatory cytokines generated and released from pancreatic islet infiltrating immune cells. In sensible combinations, which differ from such therapies in other autoimmune diseases, they represent the most powerful therapeutic tools available so far to reverse autoimmunity and to normalize diabetic hyperglycaemia in the T1D scenario. An additional advantage is that combination therapies allow the minimization of the amounts of the individual antibody components which are effective in reversing diabetic hyperglycaemia while at the same time minimizing the risk of a significant anti-TCR mediated lymphocyte reduction in the circulation [29].

## Supplementary information

Additional file 1: Supplementary information including Tables S1-S4. Table S1. Primary antibodies with their dilutions used for immunohistochemistry. Table S2. Polyclonal secondary antibodies with their dilutions used for immunohistochemistry and for in situ RT-PCR. Table S3. Sequences of primers used for in situ RT-PCR. Table S4. Ratio of proliferation/apoptosis for pancreatic β cells in islets from control and treated IDDM rats. All values for the β cell proliferation rate and apoptosis rate of the animals treated with anti-TCR (0.5 mg/kg b.wt. for 5 days) in combination with IL-6 (0.01 mg/kg b.wt. for 5 days) or with IL-17 (0.1 mg/kg b.wt. for 5 days) or in triple fashion before, immediately after and 60 days after the end of different therapies were shown in Fig. 3a, b. Data are mean values ± SEM. (DOCX 20 kb)

Additional file 2: **Figure S1.** Effects of monotherapies and the combination of anti-IL-6 plus anti-IL-17 on the metabolic profile of IDDM rats after diabetes manifestation. a-d Blood glucose concentration (mmol/l) changes are shown for the non-responding rats in response to the different therapies a with anti-TCR (0/4), b anti-IL-6 (0/4), c anti-IL-17A (0/4) or d in the combination anti-IL-6 plus anti-IL-17a (0/4). The first dashed line at day 0 indicates the start of therapy (1st biopsy) and the second dashed line at day 5 indicates the end of therapy (2nd biopsy). e Serum C-peptide concentration changes (pmol/l) are shown for rats non-responding to the different therapies. Data are mean values ± SEM. Comparison of the different experimental groups by one way ANOVA followed by Bonferroni test ***p < 0.001, to the healthy control, ^$^*p* < 0.05 to mono therapy anti-IL-6, ^§^*p* < 0.05 to mono therapy anti-IL-17A, ^^^^*p* < 0.01 to mono therapy anti-TCR and ^##^*p* < 0.01 to combination anti-IL-6 plus anti-IL-17A for each observation time point. (TIF 555 kb)

Additional file 3: **Figure S2.** Relation between initial blood glucose concentration and β cell mass after end of therapy. a after mono therapy with anti-TCR, b with anti-IL-6, c with anti-IL-17A or d the combination with both cytokine antibodies**.** The β cell mass was < 0.5 mg after all treatments. (TIF 315 kb)

Additional file 4: **Figure S3.** Morphometric analyses of β cells and immune cells in IDDM rats after monotherapies and combination of both cytokine antibodies. Changes in the rate of a proliferation, b apoptosis, c islet infiltration score and d pancreatic β cell mass after mono therapy with anti-TCR, with anti-IL-6, with anti-IL-17A or with the combination of both cytokine antibodies after diabetes manifestation. Measurements were performed immediately before therapy (1st biopsy), at the end of therapy (2nd biopsy) and 60 days after the end of therapy. Data are mean values ± SEM. Comparison of the different experimental groups by one way ANOVA followed by Bonferroni test ***p < 0.001, **p < 0.01 and *p < 0.05 to the healthy control, ^$$$^*p* < 0.001 and ^$$^*p* < 0.01 to mono therapy anti-IL-6, ^§§§^*p* < 0.001 and ^§^*p* < 0.05 to mono therapy anti-IL-17A, ^^^^^*p* < 0.001 and ^^^^*p* < 0.01 to mono therapy anti-TCR and ^###^*p* < 0.001, ^##^*p* < 0.01 and ^#^*p* < 0.05 to combination anti-IL-6 plus anti-IL-17A for each observation time point. Numbers of pancreases analysed as given in Additional file 2: Figure S1. (TIF 690 kb)

## Data Availability

All relevant data analysed during the current study are within the paper and its additional file.

## Abbreviations

Anti-TCR antibody: Anti-T cell receptor antibody
IDDM rat: LEW.1AR1-*iddm* rat
IFN-γ: Interferon gamma
IL: Interleukin
RT-PCR: Reverse transcriptase-polymerase chain reaction
TNF-α: Tumour necrosis factor alpha
