# Supplementary material for: Remission of autoimmune diabetes by anti-TCR combination therapies with anti-IL-17A or/and anti-IL-6 in the IDDM rat model of type 1 diabetes
Source: BMC Med. 2020 Feb 28;18:33. doi: 10.1186/s12916-020-1503-6 (PMC7047363; doi:10.1186/s12916-020-1503-6)
Supplement: Supplementary file 1 — Supplementary information including Tables S1-S4. Table S1. Primary antibodies with their dilutions used for immunohistochemistry. Table S2. Polyclonal secondary antibodies with their dilutions used for immunohistochemistry and for in situ RT-PCR. Table S3. Sequences of primers used for in situ RT-PCR. Table S4. Ratio of proliferation/apoptosis for pancreatic β cells in islets from control and treated IDDM rats. All values for the β cell proliferation rate and apoptosis rate of the animals treated with anti-TCR (0.5 mg/kg b.wt. for 5 days) in combination with IL-6 (0.01 mg/kg b.wt. for 5 days) or with IL-17 (0.1 mg/kg b.wt. for 5 days) or in triple fashion before, immediately after and 60 days after the end of different therapies were shown in Fig. 3a, b. Data are mean values ± SEM. (DOCX 20 kb) [file 12916_2020_1503_MOESM1_ESM.docx]

**Additional file 1:** Supplementary information including tables S1-S4

**Table S1.** Primary antibodies with their dilutions used for immunohistochemistry

| Peptide/protein  Target | Clone | Manufacturer, catalog #,  and/or name of individual  providing the antibody | Species raised in  monoclonal or  polyclonal | Dilution  used |
| --- | --- | --- | --- | --- |
| Insulin |  | DAKO; A0564 | guinea pig; polyclonal | 100 |
| Insulin | D3E7 | Bio-Rad, 5330-3369G | mouse; monoclonal | 600 |
| GLUT2 |  | Bio-Rad; 4670-1659 | rabbit; polyclonal | 1000 |
| CD8α | OX-8 | Bio-Rad; MCA48R | mouse; monoclonal | 100 |
| CD8ß | 341 | Bio-Rad; MCA938 | mouse; monoclonal | 100 |
| CD68 | ED1 | Bio-Rad; MCA341R | mouse; monoclonal | 100 |
| Ki 67 |  | Acris; AP00606PU-N | rabbit; polyclonal | 400 |
| CD3 | 1F4 | Bio-Rad; MCA772GA | mouse; monoclonal | 100 |
| CD4 | W3/25 | Bio-Rad; MCA55G | mouse; monoclonal | 100 |
| IL-1β |  | Bio-Rad; AAR15G | rabbit; polyclonal | 100 |
| IFN-γ | DB-1 | Bio-Rad; MCA1301 | mouse; monoclonal | 400 |
| TNFα |  | Bio-Rad; AAR33 | rabbit; polyclonal | 400 |
| IL-2 |  | R&D-Systems; AF-502-NA | goat; polyclonal | 200 |
| IL-10 |  | Bio-Rad; AAR29 | rabbit; polyclonal | 100 |
| IL-17A (H-132) |  | Santa Cruz Biotechnology; sc-7927 | rabbit; polyclonal | 100 |
| IL-17A |  | LSBio; LS-B13072 | rabbit; polyclonal | 200 |
| IL-6 |  | PeproTech; 500-P73G | goat; polyclonal | 200 |
| IL-4 |  | Bio-Rad; AAR16G | rabbit; polyclonal | 200 |

**Table S2.** Polyclonal secondary antibodies with their dilutions used for immunohistochemistry and for *in situ* RT-PCR

| Species/  Target | Label | Manufacturer, catalog #,  and/or name of individual  providing the antibody | Species raised in | Dilution  used |
| --- | --- | --- | --- | --- |
| Guinea pig | Alexa Fluor 488 | Dianova; 106-546-003 | goat | 400 |
| Goat | Cy 3 | Dianova; 705-166-147 | donkey | 400 |
| Goat | Cy 3 | Dianova; 305-166-045 | rabbit | 400 |
| Mouse | DyLight 488 | Bio-Rad; STAR117D488GA | goat | 400 |
| Mouse | Cy 3 | Dianova; 315-165-047 | rabbit | 400 |
| Rabbit | Cy 3 | Dianova; 111-165-047 | goat | 400 |
|  |  |  |  |  |
| Guinea pig | Biotin | Dianova; 106-066-003 | goat | 1000 |
| Goat | Biotin | Dianova; 305-065-006 | rabbit | 1000 |
| Mouse | Biotin | Dianova; 115-065-068 | goat | 1000 |
| Rabbit | Biotin | Dianova; 111-065-144 | goat | 1000 |
|  |  |  |  |  |
| Digoxigenin | unconjugated | Vector Laboratories; MB-7000 | goat | 200 |
| Goat | Biotin | Vector Laboratories; AK-5005 | rabbit | 200 |
|  |  |  |  |  |

**Table S3.** Sequences of primers used for *in situ* RT-PCR

| Gene | Accession Number | F (forward)  R (reverse) | Primer sequence |
| --- | --- | --- | --- |
| *Actb* | NM_031144 | F  R | 5’-ACAGCTGAGAGGGAAATCGT-3’  5’-CTGCTTGCTGATCCACATCT-3’ |
| *Il1b* | NM_031512 | F  R | 5’-GATGTTCCCATTAGACAGCTGCACTG-3’  5’-CTTTTCCATCTTCTTCTTTGGGTATTGT-3’ |
| *Ifng* | NM_138880 | F  R | 5’-GCTCTGCCTCATGGCCCTCTC-3’  5’-TGTTGCTGATGGCCTGGTTGTC-3’ |
| *Tnf* | X66539 | F  R | 5’-CTACTGAACTTCGGGGTGATCGGTC-3’  5’-CTGGTATGAAGTGGCAAATCGGCT-3’ |
| *Il2* | NM_053836 | F  R | 5‘-TGGAGCAGCTGTTGCTGGAC-3‘  5‘-TGGCTCATCATCGAATTGGCACT-3‘ |
| *Il6* | NM_012589 | F  R | 5‘-CCAGTATATACCACTTCACAAGTCGGA-3‘  5‘-CAAGATGAGTTGGATGGTCTTGGTC-3‘ |
| *Il17* | NM_001106897 | F  R | 5‘-GGTACTCATCCCTACAAGTTCA-3‘  5‘-CTCTTCAGGACCAGGATCTCTT-3‘ |
| *Il4* | X16058 | F  R | 5’-TCTCAGCCCCCACCTTGCTG-3’  5’-TTGCGAAGCACCCTGGAAGC-3’ |
| *Il10* | NM_012854 | F  R | 5’-TGCACCCACTTCCCAGTCAGC-3’  5’-CACCTGCTCCACTGCCTTGC-3’ |

**Table S4.** Ratio of proliferation/apoptosis for pancreatic β cells in islets from control and treated IDDM rats.

|  |  | combination of anti-TCR with | |  |  |
| --- | --- | --- | --- | --- | --- |
| Time of Therapy | Healthy control | anti-IL-6 | anti-IL-17 | anti-IL-6 + anti-IL-17 | Diabetic control |
| Before |  | 0.97 ± 0.17 | 1.18 ± 0.16 | 0.98 ± 0.05 |  |
| After |  | 1.47 ± 0.40 | 2.05 ± 0.27 | 1.51 ± 0.43 |  |
| 60 days without | 4.28 ± 0.94 | 1.79 ± 0.38 | 1.97 ± 0.43 | 2.80 ± 0.62 | 1.35 ± 0.14 |

All values for the β cell proliferation rate and apoptosis rate of the animals treated with anti-TCR **(**0.5 mg/kg b.wt. for 5 days) in combination with IL-6 (0.01 mg/kg b.wt. for 5 days) or with IL-17 (0.1 mg/kg b.wt. for 5 days) or in triple fashion before, immediately after and 60 days after the end of different therapies were shown in Fig. 3a, b. Data are mean values ± SEM.
