# Supplementary figures and images for: Remission of autoimmune diabetes by anti-TCR combination therapies with anti-IL-17A or/and anti-IL-6 in the IDDM rat model of type 1 diabetes
Source: BMC Med. 2020 Feb 28;18:33. doi: 10.1186/s12916-020-1503-6 (PMC7047363; doi:10.1186/s12916-020-1503-6)

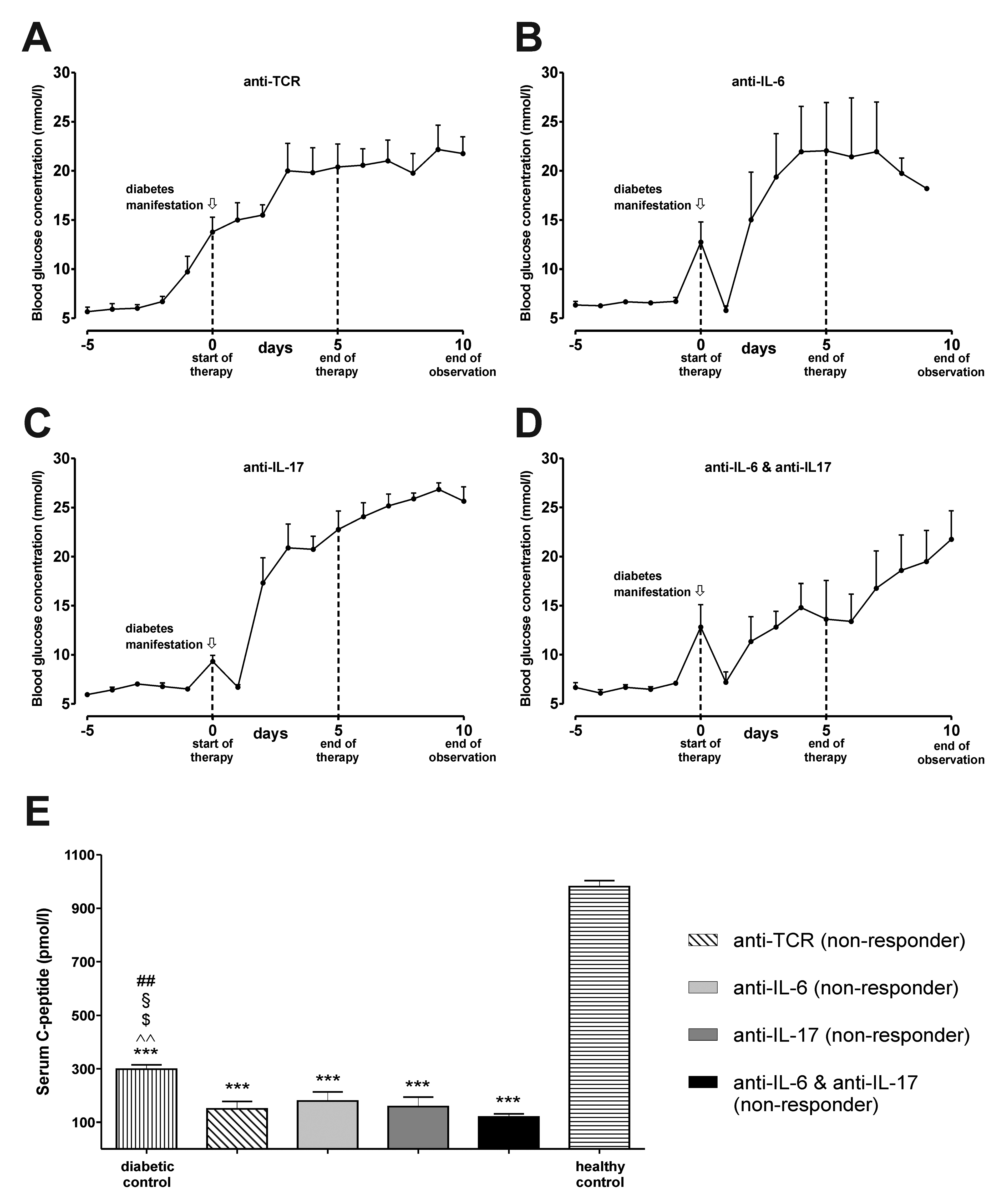

Supplement: Supplementary file 2 — Figure S1. Effects of monotherapies and the combination of anti-IL-6 plus anti-IL-17 on the metabolic profile of IDDM rats after diabetes manifestation. a-d Blood glucose concentration (mmol/l) changes are shown for the non-responding rats in response to the different therapies a with anti-TCR (0/4), b anti-IL-6 (0/4), c anti-IL-17A (0/4) or d in the combination anti-IL-6 plus anti-IL-17a (0/4). The first dashed line at day 0 indicates the start of therapy (1st biopsy) and the second dashed line at day 5 indicates the end of therapy (2nd biopsy). e Serum C-peptide concentration changes (pmol/l) are shown for rats non-responding to the different therapies. Data are mean values ± SEM. Comparison of the different experimental groups by one way ANOVA followed by Bonferroni test ***p < 0.001, to the healthy control, $p < 0.05 to mono therapy anti-IL-6, §p < 0.05 to mono therapy anti-IL-17A, ^^p < 0.01 to mono therapy anti-TCR and ##p < 0.01 to combination anti-IL-6 plus anti-IL-17A for each observation time point. (TIF 555 kb) [file 12916_2020_1503_MOESM2_ESM.tif]

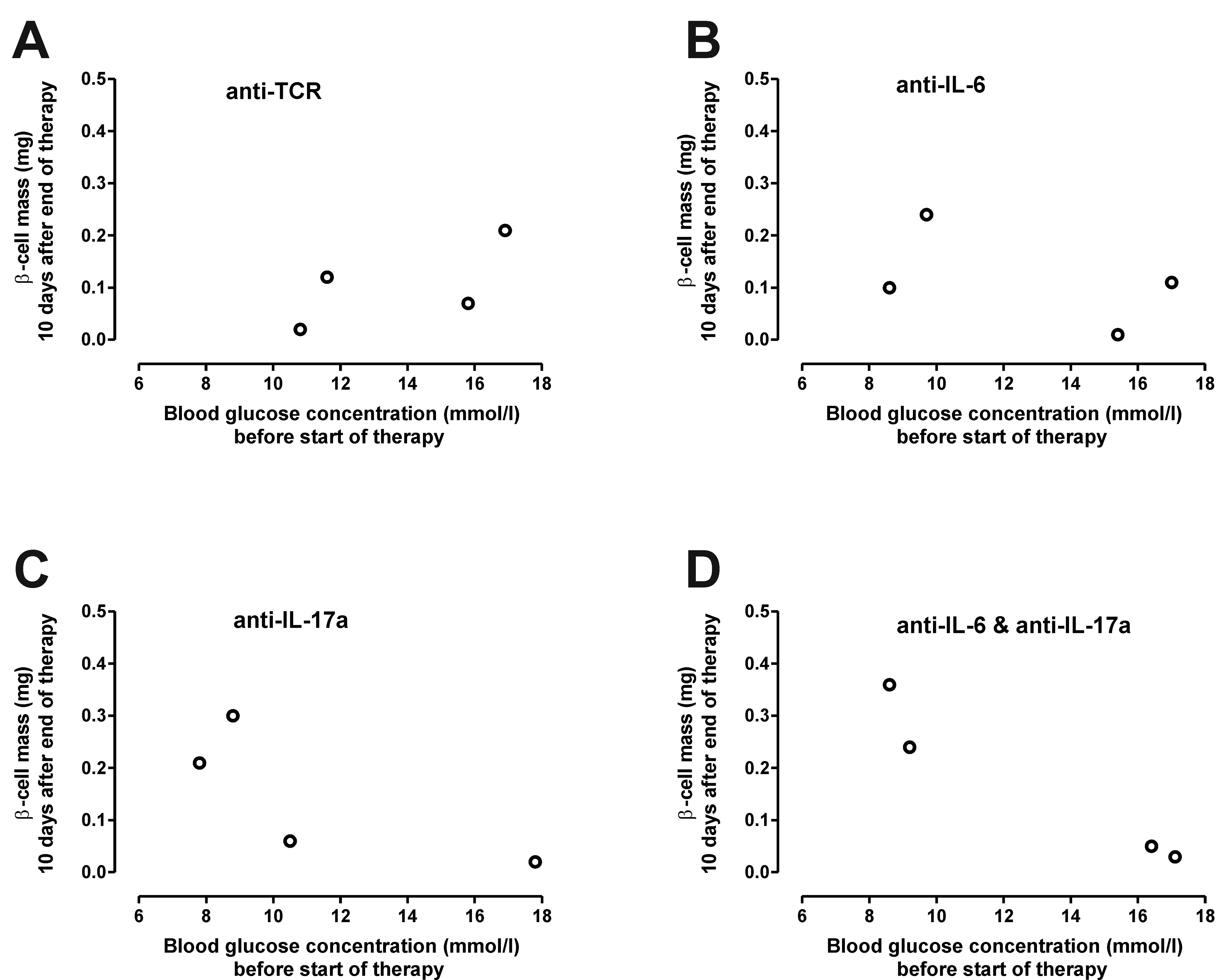

Supplement: Supplementary file 3 — Figure S2. Relation between initial blood glucose concentration and β cell mass after end of therapy. a after mono therapy with anti-TCR, b with anti-IL-6, c with anti-IL-17A or d the combination with both cytokine antibodies. The β cell mass was < 0.5 mg after all treatments. (TIF 315 kb) [file 12916_2020_1503_MOESM3_ESM.tif]

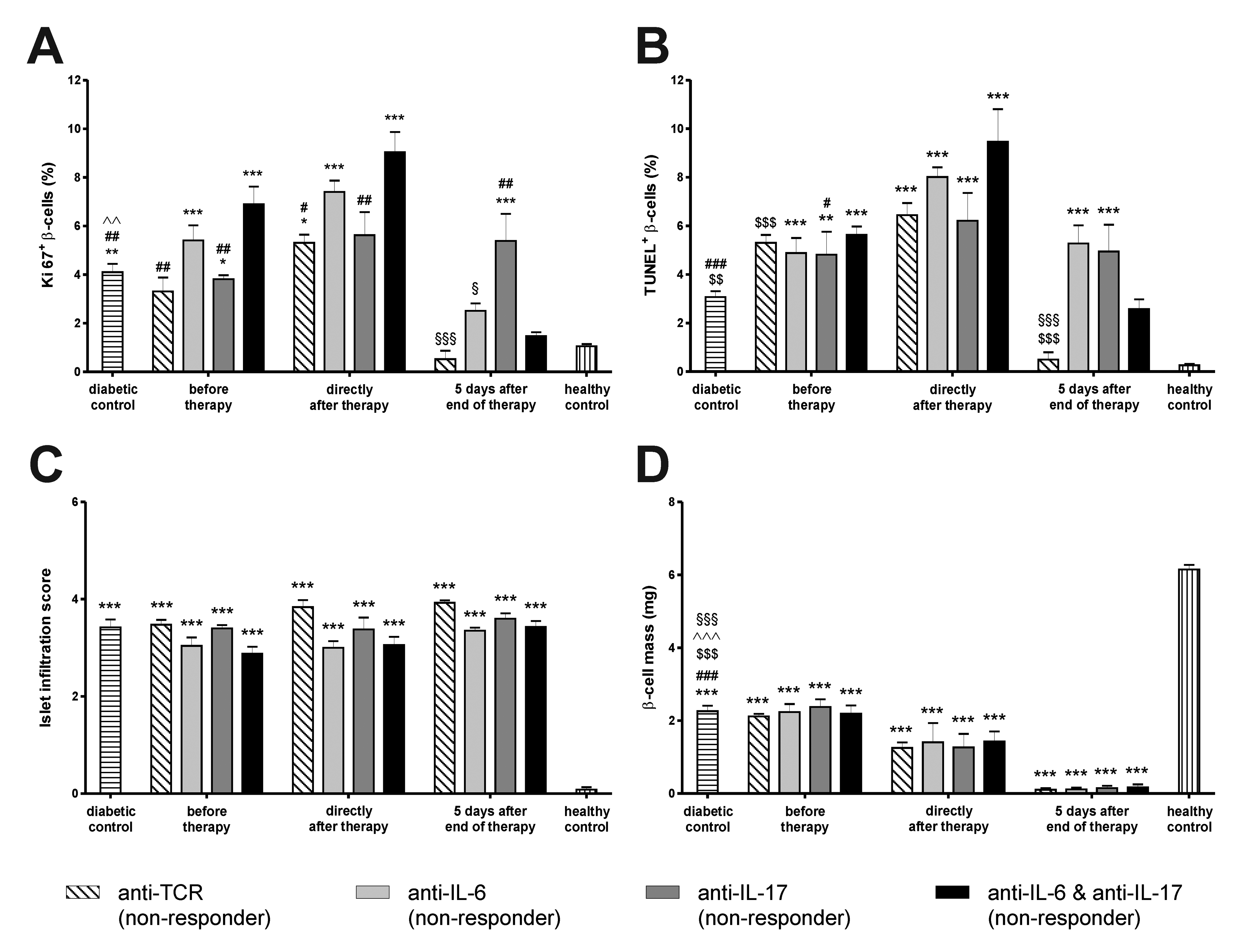

Supplement: Supplementary file 4 — Figure S3. Morphometric analyses of β cells and immune cells in IDDM rats after monotherapies and combination of both cytokine antibodies. Changes in the rate of a proliferation, b apoptosis, c islet infiltration score and d pancreatic β cell mass after mono therapy with anti-TCR, with anti-IL-6, with anti-IL-17A or with the combination of both cytokine antibodies after diabetes manifestation. Measurements were performed immediately before therapy (1st biopsy), at the end of therapy (2nd biopsy) and 60 days after the end of therapy. Data are mean values ± SEM. Comparison of the different experimental groups by one way ANOVA followed by Bonferroni test ***p < 0.001, **p < 0.01 and *p < 0.05 to the healthy control, $$$p < 0.001 and $$p < 0.01 to mono therapy anti-IL-6, §§§p < 0.001 and §p < 0.05 to mono therapy anti-IL-17A, ^^^p < 0.001 and ^^p < 0.01 to mono therapy anti-TCR and ###p < 0.001, ##p < 0.01 and #p < 0.05 to combination anti-IL-6 plus anti-IL-17A for each observation time point. Numbers of pancreases analysed as given in Additional file 2: Figure S1. (TIF 690 kb) [file 12916_2020_1503_MOESM4_ESM.tif]
